# Growth of *Caenorhabditis elegans* in Defined Media Is Dependent on Presence of Particulate Matter

**DOI:** 10.1534/g3.117.300325

**Published:** 2017-12-08

**Authors:** Matthew R. Flavel, Adam Mechler, Mahdi Shahmiri, Elizabeth R. Mathews, Ashley E. Franks, Weisan Chen, Damien Zanker, Bo Xian, Shan Gao, Jing Luo, Surafel Tegegne, Christian Doneski, Markandeya Jois

**Affiliations:** * School of Life Sciences, La Trobe University, Bundoora, Melbourne, Victoria 3083, Australia; † La Trobe Institute of Molecular Sciences, La Trobe University, Bundoora, Melbourne, Victoria 3083, Australia; ‡ Beijing Key Laboratory of Diagnostic and Traceability Technologies for Food Poisoning, Beijing Center for Disease Prevention and Control/Beijing Center of Preventive Medicine Research, 100013, China; § Key Laboratory of Computational Biology, Chinese Academy of Sciences Center for Excellence in Molecular Cell Science, Collaborative Innovation Center for Genetics and Developmental Biology, Chinese Academy of Sciences-Max Planck Partner Institute for Computational Biology, Shanghai Institutes for Biological Sciences, Chinese Academy of Sciences, 200031, China; ** Beijing Key Laboratory of Environmental Toxicology, Capital Medical University, Beijing 100069, China

**Keywords:** *Caenorhabditis elegans*, nutrition, axenic media, liposomes, feeding

## Abstract

*Caenorhabditis elegans* are typically cultured in a monoxenic medium consisting of live bacteria. However, this introduces a secondary organism to experiments, and restricts the manipulation of the nutritional environment. Due to the intricate link between genes and environment, greater control and understanding of nutritional factors are required to push the *C. elegans* field into new areas. For decades, attempts to develop a chemically defined, axenic medium as an alternative for culturing *C. elegans* have been made. However, the mechanism by which the filter feeder *C. elegans* obtains nutrients from these liquid media is not known. Using a fluorescence-activated cell sorting based approach, we demonstrate growth in all past axenic *C. elegans* media to be dependent on the presence of previously unknown particles. This particle requirement of *C. elegans* led to development of liposome-based, nanoparticle culturing that allows full control of nutrients delivered to *C. elegans*.

*Caenorhabditis elegans* is a dynamic model organism used across diverse fields such as genetics, neurobiology, biochemistry, physiology, and space exploration. Its importance is demonstrated by major roles in the awarding of six Nobel Prizes (Brenner 1974; Ellis and Horvitz 1986; Fire *et al.* 1998; Chalfie 1994). *C. elegans* are typically cultured in the laboratory on a variety of bacterial strains, with *Escherichia coli* OP50 strain the most commonly used food source. Due to the intricate link between genes and environment, greater control and understanding of nutritional factors delivered to these nematodes is required to push the field into new areas.

Differences in bacterial metabolism can alter the response of *C. elegans* to drug treatments (García-González *et al.* 2017; Scott *et al.* 2017). Therefore, inclusion of bacteria into the experimental system may introduce an unwanted variable into applications such as drug screening and toxicology. Attempts have been made to develop a bacteria-free and chemically defined culture medium (Dougherty *et al.* 1959; Vanfleteren 1974; Croll *et al.* 1977; Lu and Goetsch 1993; Clegg *et al.* 2002; Houthoofd *et al.* 2002; Szewczyk *et al.* 2003, 2006; Nass and Hamza 2007; Lenaerts *et al.* 2008). However, these axenic methods are associated with a caloric restriction state, as indicated by slower life stage progression, increased lifespan, metabolic changes, and an altered phenotype reflecting an undernourished organism (Vanfleteren 1974; Croll *et al.* 1977; Lu and Goetsch 1993; N. J. Szewczyk *et al.* 2003; Szewczyk *et al.* 2006). While these media can be prepared as solid or liquid, the majority of axenic experiments are carried out in liquid medium. This is an interesting phenomenon as the feeding behavior and physiology of *C. elegans* suggest that a liquid diet would be an inefficient delivery method of nutrients to the worms. Theoretically, no exclusively liquid diet should be able to provide nutrients to *C. elegans*, as they are filter feeders that actively eject liquid and particles smaller than bacteria, while trapping larger particles for ingestion (Fang-Yen *et al.* 2009).

One of the axenic media options available is a semi-defined medium referred to in this article as AXM. This preparation consists of soy peptone, yeast extract, and hemoglobin (Houthoofd *et al.* 2002). This medium is known to induce a caloric restriction state, despite a high concentration of calories being present in the medium (Houthoofd *et al.* 2002; Lenaerts *et al.* 2008; Greer and Brunet 2009). This also limits research to topics concerning dietary restriction, and the semi-defined nature of the medium restricts the understanding and manipulation of the nutrient concentrations delivered to the worm.

Some media, such as *C. elegans* Habituation and Reproduction (CeHR) medium, require milk supplementation, but milk is included without understanding its role in nematode nutrition (Clegg *et al.* 2002). In this protocol, milk makes up 20% of the final volume of the medium; therefore, the inclusion of milk may introduce a unique set of issues to researchers, especially those interested in nutrition. The nutritional composition of milk varies due to a range of factors, and it is a complex and poorly understood matrix (Haug *et al.* 2007). Due to the inclusion of milk or milk components, the medium could be considered as semi-defined, as the precise concentrations of nutrients are unknown.

While CeHR axenic medium includes a large volume of milk supplementation, milk-free alternatives are available. One example is a defined medium known as *C. elegans* Maintenance Medium. However, worms in this medium are slow to develop and lay eggs taking 7 d to reach 1 mm and 9 d to lay eggs (Szewczyk *et al.* 2003). While slow, this development, which occurs in the absence of milk supplementation, provides an interesting opportunity to explore whether there is a common factor in both these media that is required to support growth.

These limitations have prohibited the widespread uptake of axenic media for *C. elegans* experiments. It has been hypothesized the dietary restriction may be due to a component or a growth factor present in bacteria, but lacking in axenic medium recipes (Vanfleteren 1974; Lu and Goetsch 1993; Lenaerts *et al.* 2008). Therefore, a central aim of our investigation was to further the understanding of the critical factors involved in successful axenic *C. elegans* culture, in order to overcome some of the limitations that have restricted the application of these methodologies.

## Materials and Methods

### C. elegans strains and conditions for growth rate experiments

Wild-type Bristol N2 (Caenorhabditis Genetics Center) were used in all experiments. All experiments were conducted at 20° and kept away from light to prevent photodegradation of light-sensitive components present in media. Prior to growth rate assays, worms were cultured on standard NGM medium, with living OP50 *E. coli* as food source. Synchronized eggs from these colonies were hatched in M9 buffer solution, and added to differing media conditions at life stage L1. Single worms were selected randomly and added to each well of a 96 well-plate, preloaded with 150 μl of culture medium as treatment; 25 worms per treatment were measured at 24-hr intervals, and results presented are averages with SD. All growth rate experiments were carried out in triplicate. Images were analyzed using FIJI (Image J) software, with length measurements being derived by the sum-total of a number of segmented lines of known length, down the center of the worm. OP50 *E. coli* were prepared at 1× and 5× concentrations in S-medium as positive controls to bacterial cells per milliliter of ∼9 × 10^8^ and ∼4.5 × 10^9^, respectively. Animals were excluded from analysis if fungal or bacterial contamination was detected.

### Media preparation

A variety of axenic media preparations were used. The majority of these media were based predominantly on CeHR medium; however, in many cases, we have made moderations to the original protocol (Clegg *et al.* 2002). To clarify these modifications, when we refer to “CeHR + Milk” this medium follows the usual published protocols to prepare CeHR medium. In preparations referred to as “CeHR + Milk Particulate Matter,” the milk was centrifuged at 10,000 × *g* for 30 min to ensure distinct separation between pellet and supernatant. The remaining pellet of milk was resuspended to the initial volume of whole milk with M9 buffer and added to the CeHR medium, making up 20% of the final volume of media. Growth rate was also measured in worms grown exclusively in buffer resuspended milk pellet (referred to in results as “Milk Particulate matter”). Media referred to as “CeHR no milk” followed the usual protocols for CeHR preparation; however, at the stage of the protocol where milk is usually added, it was replaced with the same volume of deionized water. This made up 20% of final volume in order to approximate the same concentration of solutes in the usual CeHR preparation, while removing the effect of the milk. Growth rate in M9 was also included as a negative control, because there is no nutrient included in the mix and L1 arrest is expected, except in the event of contamination. CeMM media was prepared by Cell Guidance Systems (Cambridge, UK), following the usual protocol (Szewczyk *et al.* 2003). AXM media was prepared in our laboratory, following the usual protocol (Lenaerts *et al.* 2008). All filtrations were conducted using an 0.22 μm Stericup Vacuum filter unit (Millipore).

### Solubilization of particulate fraction in milk

Ultraheat-treated (UHT) skim milk was centrifuged at 10,000 × *g* for 30 min. The supernatant was discarded, leaving only the milk pellet. High concentration urea (8 M) was slowly added and mixed with the pellet until the sample had changed from an opaque white color to a relatively translucent mixture, and the volume of added urea recorded. This mixture was again diluted back to the original volume of milk using M9 buffer and then added to the CeHR mixture. It was assumed that the solubilized proteins would still be available to the worm; however, they would be in solution rather than as particles. We chose to include a control against the effect of urea at a concentration of 46 mM. For this control we prepared CeHR + Milk Particulate Matter medium as described in the previous section, but added urea to a final concentration of 46 mM included in the medium. As 8 M urea applied directly to the milk pellet was required to solubilize the proteins in the treatment medium, it was assumed that, in this control, protein particles would not be in solution, and would remain as formed particles.

### DNA extraction and qualitative polymerase chain reaction

Replicates (50 ml) of UHT milk samples were centrifuged at 10,000 × *g* for 30 min. The supernatant was discarded, leaving only the milk pellet. Milk pellet was weighed into replicates of 0.34 g mean weight. MoBio PowerSoil DNA Isolation Kit (Qiagen) was utilized to extract gDNA following the manufactures instructions. Extractions were performed in triplicate, and total gDNA concentration was assessed using Implen Nanophotometer P330.

### Qualitative analysis of bacterial DNA in sample

The intergenic spacer region (IGS) between the bacterial 16S and 23S rRNA subunits was amplified as a qualitative assessment of bacterial gDNA presence in the UHT milk. The forward primer 16S-1392F 5′FAM-GYACACACCGCCCGT-3′ and reverse primer 23S-125R 5′-GGGTTBCCCCATTCRG-3′, often utilized for automated method of ribosomal intergenic spacer analysis, were used for amplification (Weisburg *et al.* 1991). PCR reactions were performed using TopTaq reagents (Qiagen), and contained 2 μl TopTaq 10× buffer, 4 μl Q solution, 1.2 μl MgCl_2_, 1 μl dNTPs (10 mM), 1 μl of 10 µM forward and reverse primers, 0.1 μl TopTaq DNA polymerase, 8.7 μl sterile MilliQ water (sddH_2_O) and 1 μl of sample DNA template per 20 μl reaction. Reactions were performed using T-Professional TRIO Thermalcycler (Biometra) with an initial denaturation at 94° for 180 sec, 35 cycles of denaturation at 95° for 60 sec, annealing at 52° for 60 sec, extension at 72° for 90sec, and a final extension at 72° for 360 sec (Kovacs *et al.* 2010). Once completed, PCR products were visualized on a 2% agarose electrophoresis gel. Wastewater samples, normalized to 5 ng/μl gDNA, acted as controls for DNA amplification.

### Quantification of bacterial DNA using real-time quantitative PCR (qPCR)

Quantitative PCR (qPCR) was used to quantify the copy number of total bacterial gDNA present in three biological replicates of the UHT milk sample. The primer pair 1114F 5′-CGGCAACGAGCGCAACCC-3′ and 1275R 5′-CCATTGTAGCACGTGTGTAGCC-3′ was used to target the bacterial 16S rRNA gene as previously described (Denman and McSweeney 2006). qPCR was performed on the CFX Connect Real-Time PCR Detection System (Bio-Rad), and each sample was loaded in triplicate. Each 20 µl reaction contained 3.3 µl SsoAdvanced Universal SYBR Green Super Mix, 0.27 µl of each 10 µM forward and reverse primer, 14.16 µl sterile MilliQ water (sddH_2_O), and 2 µl of sample gDNA. The PCR product was run on 1.5% (w/v) agarose gel and purified using QIAquick Gel Extraction Kit (Qiagen). Bacterial copy number was calculated for a 130-bp amplicon using the 1114F and 1275R primer pair to amplify the 16S rRNA gene in purified *E. coli* strain DH5α. The standard curve was generated using 10-fold serial dilutions of purified product, in triplicate. The cycle settings for the qPCR were: 94° for 180 sec, followed by 40 cycles of 94° for 10 sec and 60° for 30 sec.

### FACS analysis for particle detection

Aliquots (20 μm) of a variety of media preparations and conditions were analyzed for the presence of particulate matter. These included CeHR supplemented with milk, CeHR filtered, CeMM, CeMM filtered, CeMM filtered and then incubated at 20° for 72 hr, AXM media, AXM media filtered, and AXM media filtered and then incubated for 72 hr at 20°. FACS analysis was performed on the CytoFLEX S FACS machine (Beckman Coulter), using a 405 nm violet laser side scatter channel to determine particle size. 1, 0.5, and 0.05 μm. Fluoresbrite BB Carboxylate microspheres (Polysciences) were used as standards to determine size reference values to compare particles detected within samples.

### Liposome preparation and encapsulation of media

DMPC (1,2-dimyristoyl-sn-glycero-3-phosphocholine) was purchased from Avanti Polar Lipids (Alabaster, AL). Lipids were dissolved in chloroform to form a stock solution and stored at −20°. Aliquots were then measured at 20° into glass test tubes and mixed with chloroform to produce a lipid concentration of 10 μM per tube. The chloroform was evaporated under a gentle stream of nitrogen gas upon continuous vortexing to form a thin film of lipid on the wall of the test tube. These lipid tubes were then dried overnight. To encapsulate the media in liposomes, 2 ml of medium was added to a tube of dry lipid. The tube was then incubated at 37° for 30 min. This was followed by gentle vortexing to form liposomes. The 2 ml of suspended-liposome medium was then dialyzed against 1 liter of M9 buffer. This ensured that the nutrient source was contained only within the liposomes and not in the surrounding medium.

### Life stage scoring

Each worm image captured was scored for life stage. A combination of factors were taken into consideration to determine life stage. These included sign of larval molts, development of morphological features, such as gonads and vulva, or appearance of eggs/offspring; 25 worms were scored per treatment, and the life stage that represented the majority (>50%) of animals was reported.

### Statistical analysis

Statistical analysis was performed on all dietary conditions, with results presented in Supplemental Material, Table S2. Multivariate analysis was conducted using SPSS (IBM) software, with dietary condition as the fixed factor, and mean lengths and growth rates at respective time intervals selected as dependent variables.

### Data availability

No additional raw data exists that is not expressed in figures, tables or supplementary information. Reagents used are available on reasonable request.

## Results and Discussion

Initially, we used the dependence of milk supplementation in CeHR medium to address the fundamental requirements of *C. elegans* feeding. We hypothesized that the function of milk would be due to a single or combination of the following possibilities: (1) UHT skim milk may provide a source of bacterial contamination; (2) UHT skim milk may be a source of an otherwise excluded, but essential nutrient; or (3) UHT skim milk may provide a particulate, nutrient delivery vessel of media inside the worm.

**Figure 1:**
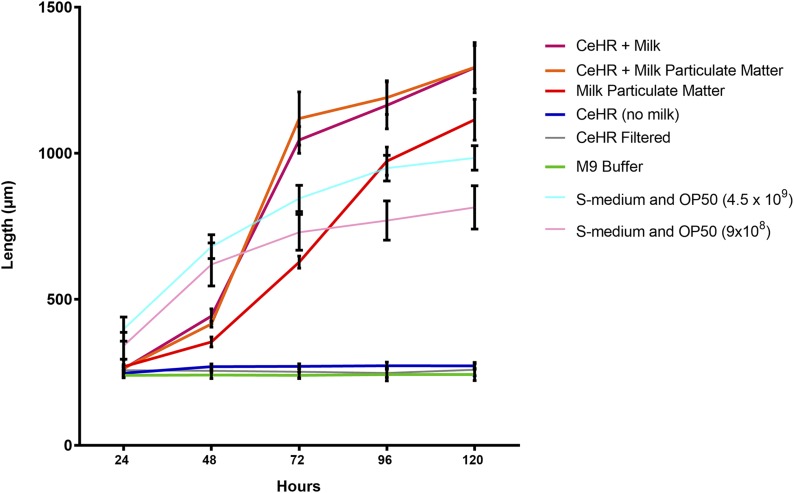
Effect of milk separation on growth rate: growth rate (length micrometer) of *C. elegans* measured at 24-hr intervals, while exposed to various dietary conditions. SD expressed as error bars. *n* = 25 per treatment group, growth in S– Medium containing OP50 *E. coli* (9 × 108 and 4.5 × 109 colonies per milliliter) included as positive control.

The hypothesis that milk provided an otherwise excluded, but essential nutrient to the medium was investigated by analysis of modified versions of CeHR medium. Initially, this involved separating suspended particulate matter from low fat, UHT milk by centrifugation and resuspending particulate matter in M9 buffer. This resuspended particulate matter was fed as a food source or used to supplement CeHR instead of whole milk. All conditions where milk particulate matter was present developed *C. elegans* to adulthood, including when only milk particulate matter was available to nematodes (Figure 1). CeHR supplemented with either milk, or milk particulate matter had the fastest growth rate (Table S2). Conditions where either milk or milk particulate matter was added to CeHR and not processed further were significantly different in total growth between 24 and 120 hr from all other related conditions (Table S2). However, adding the milk pellet only was not significantly different to including skim milk without further processing. This suggests that the isolated particulate material performs a function comparable to that of whole milk.

This growth rate could be arrested by exposure to medium filtered at 0.22 µm. Nematodes cultured in CeHR without any milk-based supplementation also entered L1 arrest—a life stage that is linked directly to nutrient availability (Baugh 2013). Both of these conditions contain an abundance of nutrients; however, they were not significantly different in total growth from the M9 control, which was used as a negative control due to its lack of nutrients (Table S2). This suggests that a lack of nutrient delivery may be the cause of the L1 arrest. Our results demonstrate that the nematode requires a component within the UHT skim milk and/or milk particulate matter. This component may have a function to assist the worms to access the nutrients, such as particulate matter, within CeHR growth medium, or may be a particulate food source, such as would be introduced by bacterial contamination.

Our finding that feeding 0.22 μm filtered CeHR medium was capable of arresting growth at L1 life stage narrowed the search for essential components in the medium to those blocked by filters of this pore size (Table S1). Filtration at this pore size is used routinely to remove bacteria. We hypothesized that UHT skim milk could cease to initiate worm development after filtration due to bacterial cell removal. The protocol states milk should be UHT; a process likely to kill all bacterial cells. However, it was unclear whether dead cells remained in the milk. Dead *E. coli* are acknowledged to initiate worm development, while reflecting a mild dietary restriction in comparison to axenic media (Greer and Brunet 2009).

Culturing of organisms found in milk only detects viable microorganisms, but does not account for nonviable bacterial cells potentially acting as a nutrition source. Molecular techniques are a useful measure of intact bacterial cells, both live and dead, present due to including milk in the medium. By detecting residual bacterial DNA in the milk, an assessment of whether bacterial cells may be present as a food source can be made. A limitation to using a molecular approach is the only nucleic acids are being detected, and no information regarding if they are still contained within a cell is provided.

We attempted to extract genomic DNA and amplify the intergenic spacer region between the 16S and 23S rRNA genes by PCR as a qualitative analysis for bacterial presence in the sample. However, bacterial DNA failed to amplify from the milk samples in PCR and no bands were observed when the PCR product was run on 2% agarose. To confirm this observation, qPCR was used to detect 16S rRNA copy number in the milk samples. Very low copy numbers were detected in the milk samples across all three biological replicates, the highest being 52.9 ± 9.7% copies per milligram of milk pellet (Table S3). In addition, CeHR medium was streaked onto NGM agar and incubated for 48 hr at 37° without any bacterial growth observed. From these results, it is reasonable to conclude that microorganisms are not acting as a major growth component of the UHT milk supplemented CeHR medium. The milk is unlikely to induce worm development due to bacterial contamination, as the presence of bacterial DNA does not guarantee that intact and edible bacterial cells are present. In the unlikely event that bacterial cells have survived the ultraheat treatment process, the low copy number values detected indicates that there would not be sufficient cells present to rival the developmental performance of worms grown on a lawn of live OP50 bacteria.

The *C. elegans* dependence on particulate matter present on milk led us to question whether this was due to a nutritional or physical factor. Casein micelles were a useful target for this analysis as they are present in milk, and could provide a protein source and/or a physical transport vessel for nutrients (Cheng *et al.* 1979; Enright *et al.* 1999; Brans *et al.* 2004). Casein micelles share a very similar range of sizes and morphology to bacteria, in addition to parallel behaviors such as aggregation (Enright *et al.* 1999).

**Figure 2:**
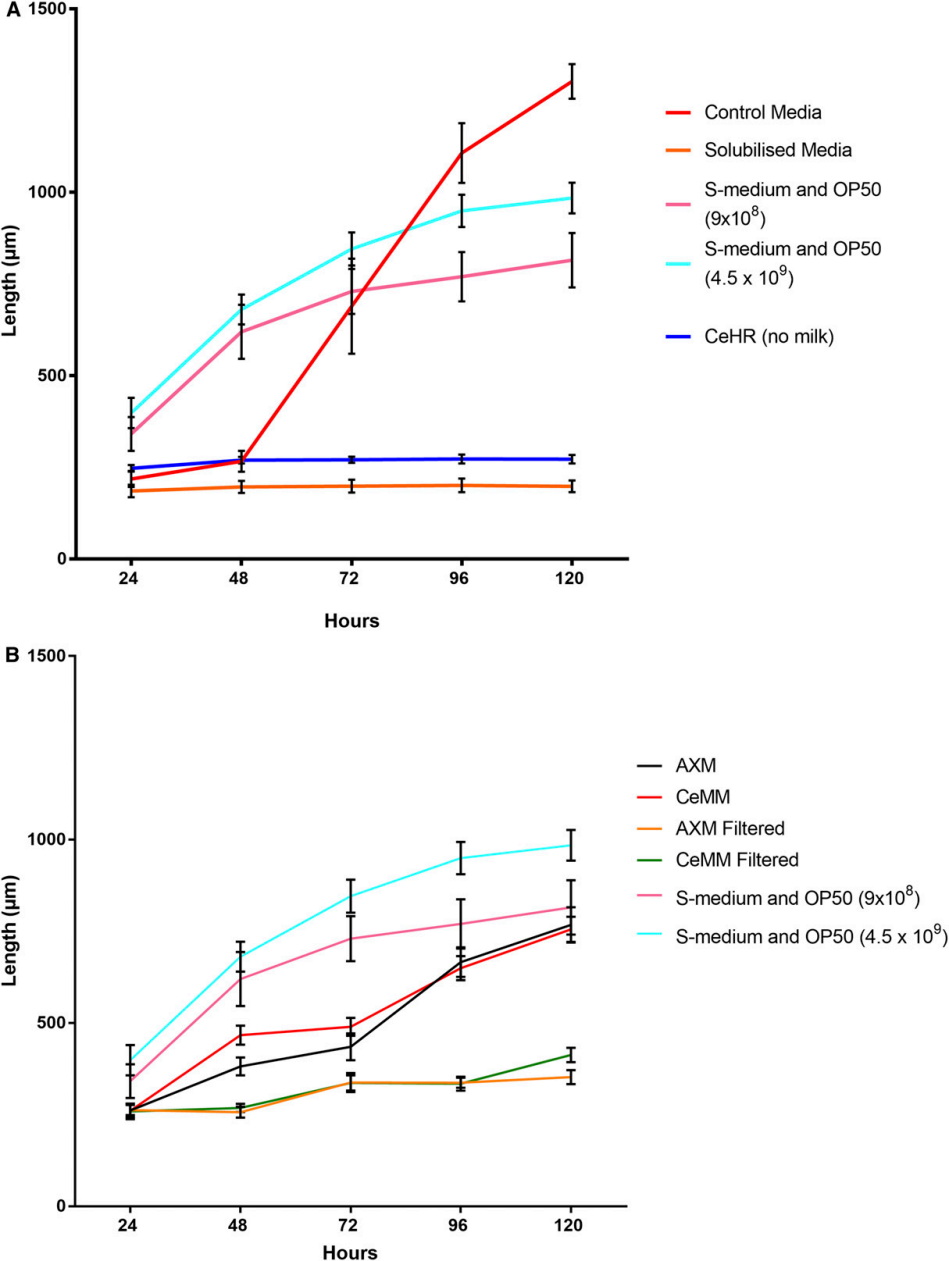
Effect of particulate matter on development: (A) Solubilization of particulate fraction in milk arrests *C. Elegans* growth. Growth rate (length micrometer) of *C. elegans* measured at 24 hr intervals, while exposed to CeHR media with added milk particulate matter that has either been solubilized with high concentration urea (8 M) and then diluted to 46 mM (solubilized media) or 46 mM urea added to CHR media + milk pellet, leaving milk pellet particles present (control media). SD expressed as error bars. *n* = 25 per treatment group. (B) Effect of filtration of AXM and CeMM media on *C. elegans* growth rate. Growth rate (length micrometer) of *C. elegans* measured at 24-hr intervals, while exposed to AXM media, CeMM media, AXM media filtered or CeMM filtered. SD expressed as error bars. *n* = 25 per treatment group, Growth in S– Medium containing OP50 *E. coli* (9 × 10^8^ and 4.5 × 10^9^ colonies per milliliter) included as positive controls in both figures.

To test whether the casein micelles were initiating worm development due to their inherent nutritional properties or by introducing particulate matter to the media, the milk pellet fraction was solubilized using 8 M urea. This maintained the nutritional composition of the pellet, while bringing all components into solution and removing particulate matter from the media. The solubilization treatment was shown to result in L1 arrest, as growth did not occur beyond the initial length of ∼200 μm (Figure 2A). The solubilized treatments total growth over the experiment was not significantly different from the M9 buffer negative control (Table S2). The control group containing equivalent quantities of urea, but also particulate matter, progressed through all life stages to adulthood. The removal of particulate matter is therefore the likely cause of arrested development. Past studies in the related nematode *Caenorhabditis briggsae* have reported the requirement for growth factors to be in a precipitated form in order to function (Vanfleteren 1974). Our results provide evidence that it is the physical presence of particulate matter from milk, rather than a specific nutrient growth factor, that is essential to *C. elegans* growth in CeHR medium.

Axenic media options that do not require milk supplementation are available to *C. elegans*, such as CeMM (Szewczyk *et al.* 2003) and AXM (Lenaerts *et al.* 2008) preparations. We sought to determine what effect, if any, particulate matter has in these media. We analyzed AXM and CeMM for developmental performance of worms fed with medium that had been subjected to 0.22 μm filtration. The protocol for CeMM includes a final step 0.22 μm filtration; however, the protocol stipulates to do so at 30° and with an hour of stirring (Szewczyk *et al.* 2003). We hypothesized that this protocol of heating and stirring may help particles to dissolve into solution and pass through the filter, before returning to their precipitated particulate form during the cooling process. To confirm our hypothesis, we followed the standard protocol, but filtered the medium an extra time immediately prior to use without heating and stirring. Using an unmodified protocol, both AXM and CeMM media initiated normal worm development (Figure 2B). However, filtering immediately before use reduced growth rate of worms fed both media types. Total growth between 24 and 120 hr was significantly less in both filtered conditions (Table S2). Using these filtered conditions,worms could be seen to be at L1 arrest or dauer life stages after 120 hr for AXM and L2 for CeMM (Table S1).

To assess whether particles were present in all media, and whether this could be correlated with worm development, we used flow cytometry (FACS) analysis for small particle detection. FACS analysis detected 8.5 × 10^6^, 6 × 10^7^ and 8 × 10^7^ particles per milliliter of CeHR, CeMM and AXM media respectively (Table 1). The number of bacteria ingested by N2 wild type C. elegans, in life stage L1–L4 has been estimated to be close to 10^5^ per worm, per day (Gomez-Amaro *et al.* 2015). All axenic media preparations contain enough particles to support the normal nematode feeding behavior, if they recognize all particles as food. Media filtration reduced the particle number to near zero levels. CeMM filtered medium showed particle number increase following incubation, indicating that this medium is prone to precipitation of components over time.

**Table 1:** Number of particles detected in tested media per milliliter

Condition	Number of Particles/ml
CeHR	8.5 × 10^6^
CeHR filtered	50
CeMM	6 × 10^7^
CeMM filtered	2.4 × 10^3^
CeMM filtered and incubated at 20° for 72 hr	1.8 × 10^4^
AXM	8 × 10^7^
AXM filtered	25
AXM filtered and incubated at 20° for 72 hr	25
Liposomes packed with CeHR	5.3 × 10^8^
Liposomes packed with M9	1.1 × 10^9^

**Figure 3:**
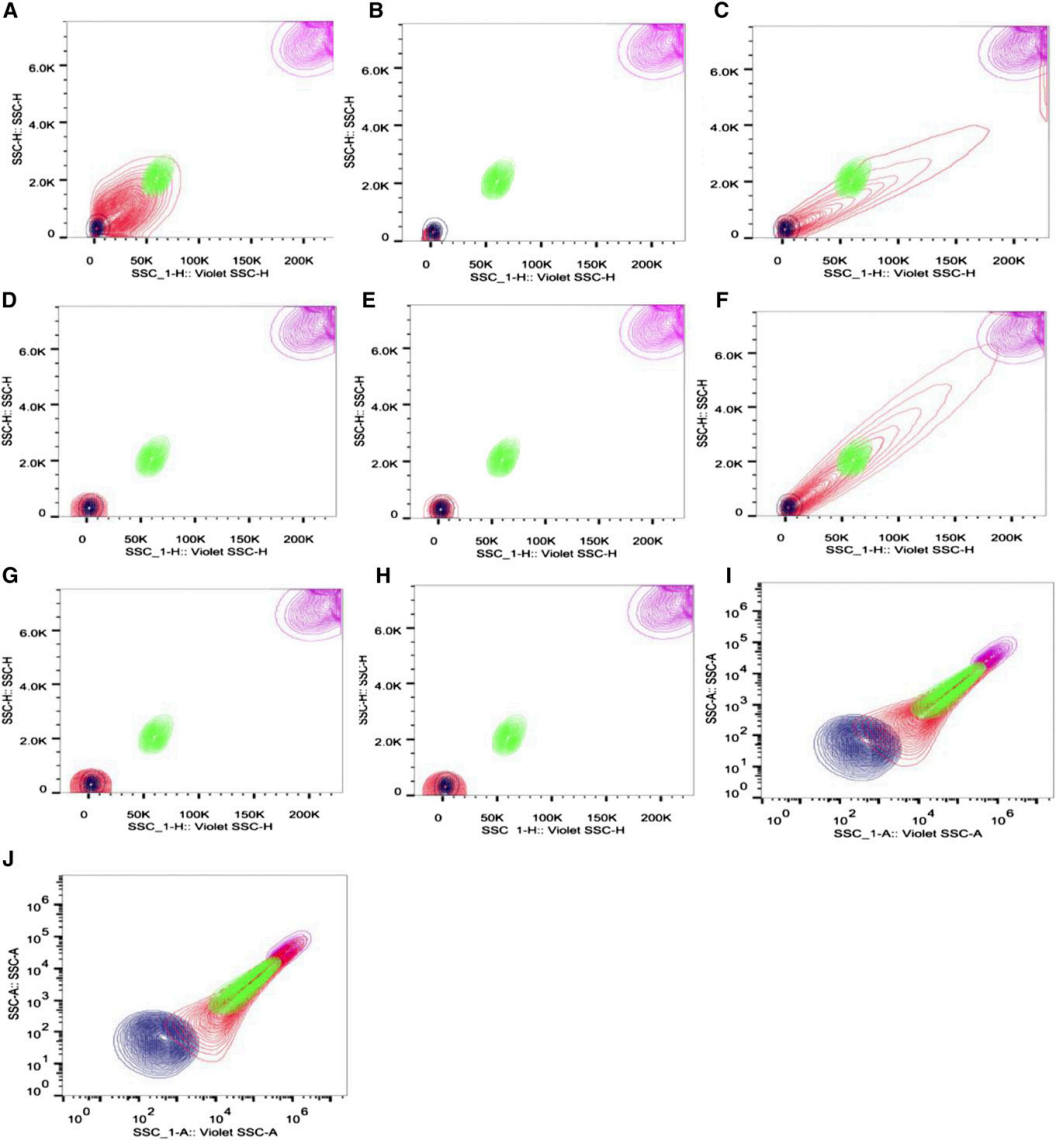
FACS detection of particles in various media conditions. Distribution of different particle sizes. Purple, 1 μm; Green, 0.5 μm; Blue, 0.05 μm; Red, sample. (A) CeHR, (B) CeHR Filtered, (C) CeMM, (D) CeMM filtered, (E) CeMM Filtered and incubated at 20°, (F) = AXM, (G) AXM Filtered, (H) AXM Filtered and incubated at 20°. (I) Liposomes packed with CeHR. (J) Liposomes packed with M9.

Particles in media filtered at 0.22 μm were detected at sizes closest to the 0.05 μm bead standards (Figure 3, B, D, E, G, and H and Table 1). The small size and number of particles across these filtered treatments correlates with poor growth performance in *C. elegans* in these conditions (Figure 1 and Figure 2B). This lack of growth indicates that smaller particles are not recognized as food, and are ejected with liquid (Fang-Yen *et al.* 2009; Kiyama *et al.* 2012). Both CeMM and AXM showed a wider distribution of particle size than CeHR, with particles detected across the range of bead size standards. CeHR, CeMM, and AXM all contain particles in high concentrations, and of a size that *C. elegans* have been demonstrated to uptake (Avery 1993; Avery and Shtonda 2003; Fang-Yen *et al.* 2009; Avery and You 2012; Kiyama *et al.* 2012).

**Figure 4:**
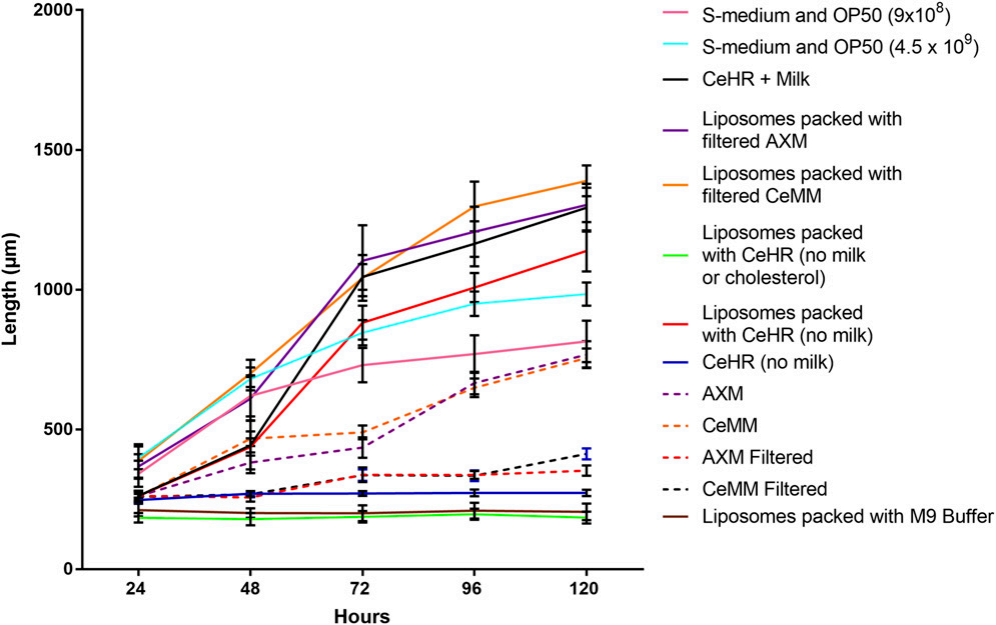
Growth rate (length micrometer) of *C. elegans* measured at 24 hr while exposed to various media conditions. CeHR, AXM, and CeMM media were both filtered following the same protocol as used in Figure 1 and Figure 2B. However, each medium was then packed inside liposomes and suspended in M9 buffer. CeHR medium with milk and cholesterol removed were also packed within liposomes and suspended in M9 buffer. M9 Buffer or CeHR without liposomes were included as negative controls. *n* = 25 per treatment group, SD presented as error bars. Growth in S-medium containing OP50 *E. coli* (9 × 10^8^ and 4.5 × 10^9^ colonies per milliliter) in addition to CeHR prepared according to standard protocols included as positive controls.

Our results indicate that worms require particles for successful nutrient uptake in axenic media. However, it is unclear what exact nutrients accompany these particles. For this reason, we sought to develop a protocol for the medium to be packaged inside artificial liposome nanoparticles. We took advantage of the relatively nondiscriminatory particle ingestion by *C. elegans* between the sizes of 0.1 and 3 μm, especially when no other food source is offered (Avery and You 2012; Kiyama *et al.* 2012). The liposomes prepared, while not being uniform in size were detected to have 3.2 × 10^8^ particles per milliliter in this size range (Figure 3). In addition, the lipid bilayer present in liposomes was selected to mimic the phospholipid bilayer present in the plasma membrane of bacteria. An added advantage of this methodology is that the *C. elegan*’*s* environment can be composed of M9 buffer following dialysis of the liposome suspension. Previous axenic medium protocols required the worm to bathe in the same medium that they use for food, adding complication to maintenance of pH, osmolarity, and preventing contamination. Growth of worms fed medium-packed liposomes, suspended in M9 buffer, also indicates that the delivery of nutrient via the liposome is effective. This is because the outside environment of the liposome does not contain nutrient. This is an important consideration for any trial testing the bioactivity of compounds, as growth in this model indicates that the compound of interest has also been delivered inside the animal. Liposomes have previously been administered to *C. elegans* to deliver compounds for longevity testing, but, to our knowledge, we are the first to successfully use them as a food delivery method in the absence of bacteria (Shibamura *et al.* 2009). Worms exposed to liposomes containing CeHR (without milk supplementation), and suspended in M9 buffer, produced significantly longer worms than *E. coli* treatments with bacterial concentrations of equivalent particle numbers, and almost an order of magnitude greater (Figure 4 and Table S2). *E. coli* treatments containing equivalent particle numbers returned significantly higher growth rates between 24 and 48 hr; however, between the next interval of 48 and 72 hr, growth rate was greater for liposomes containing nutrient (Table S2). This growth rate recovery in the second interval was observed in all CeHR conditions that led to development. It is unclear why this recovery occurs, and then exceeds total length. The need for acclimation to axenic media has been described previously, with faster growth speeds approached in future generations cultured axenically (Samuel *et al.* 2014). One possible explanation for the longer worms is the differences in nutrient content between these dietary conditions. Differences in one macronutrient, such as carbohydrate, have been observed to affect worm length (Brooks *et al.* 2009; So *et al.* 2011). These previous studies exploring the effect of nutrition on worm size analyzed the differences in nutrient between bacterial strains, whereas this investigation compares vastly different dietary profiles. Any discrepancies between the axenic diets and OP50 could reasonably result in significant growth rate differences. Liposomes now provide a tool to investigate the effect of various nutrients on growth rate, with greater control over nutrient delivery. However, until more is understood about the relationship of specific nutrients to body shape, total growth rate should not be taken in isolation as an indicator of preferable nutrition. However, the ability to initiate development using a medium that would otherwise result in L1 arrest, such as CeHR without milk added, is a significant finding. Worms fed liposome packed with M9 demonstrated L1 arrest, which suggests liposomes are not initiating development beyond acting as a mode of delivery (Table S1 and Table S2).

While the removal of particulate matter appeared to have a direct effect on worm development, it was still possible that an essential nutrient could be removed alongside the particulate matter. The evidence presented in Figure 2A suggests that the same nutrient, in a solubilized, rather than particulate form does not support development. However, this experimental design requires the inclusion of urea. The final concentration of urea in the CeHR media used in the urea experiment was calculated as 46 mM. While this concentration is well below that which *C. elegans* are reported to tolerate (up to 600 mM) (Esteban and Choe 2014), the collateral effects urea may have on the other nutrients included in the medium is unknown. However, our novel liposome nutrient delivery method was a useful tool to assess whether the removal of particulate matter was responsible for the arrested development rather than the removal of an essential nutrient. The main advantage of this method was to exclude a potentially harmful variable such as urea. Filtration at 0.22 μm of AXM and CeMM medium at room temperature was shown to arrest or result in stunted development in Figure 2B. These filtered media were then packaged within the liposome nanoparticle in an attempt to rescue development. The packaging of filtered CeMM medium in liposomes increased growth rate significantly for the first three growth intervals compared to CeMM prepared using the usual protocols, and growth rate increased significantly on the CeMM filtered medium at all growth intervals (Figure 4 and Table S2). Filtered AXM, repackaged in liposomes, increased growth speed significantly compared to both the usual protocol for AXM preparation, and when filtered immediately prior to use (Figure 4 and Table S2). In addition, both medium achieved higher average length after 120 hr compared with any other treatments in this study; however, this was a significant difference only for CeMM liposomes (Table S2). This is further evidence that liposome-based nutrient delivery is more effective than the uncontrolled and unmonitored presence of particulate matter used previously in axenic medium. In addition, if an essential nutrient had been removed by filtration, this developmental rescue and performance would not be possible unless the liposome’s chemical structure reintroduced that nutrient.

To assess the possibility that the liposome reintroduced an essential nutrient, we chose to prepare CeHR medium with an essential component missing, and observed the developmental consequences. Sterol is a lipid understood to be an essential nutrient for many nematodes include *C. elegans* (Lu *et al.* 1977). In CeHR, cholesterol serves as a defined source of lipids, and was therefore a useful candidate for exclusion. As liposomes are a lipid-based structure themselves, it was conceivable that the liposomes could mimic the nutrient function of cholesterol. In addition, sterol is added after filtration in standard CeHR preparation protocol. The protocol does not state the reason why all components of CeHR medium, except for cholesterol and milk, are filtered at 0.22 μm. It could be hypothesized that filtration of the medium may remove cholesterol. Therefore, it is important to assess whether liposomes are mimicking the action of cholesterol in the liposome model. However, as demonstrated in Figure 4, Table S1, and Table S2, the removal of cholesterol from the medium led to developmental arrest at L1 life stage, and was not significantly different to worms cultured in the M9 buffer negative control. This demonstrates that, in this context, liposomes cannot perform the nutrient role of sterol, but have the primary function of nutrient delivery. In addition, this demonstrates the potential applications of liposome-delivered nutrients in furthering the understanding of the nutrient requirements of *C. elegans*.

The liposome-based nanoparticle food reported here allows researchers to use the powerful animal model *C. elegans* to further our understanding of nutrient–gene interactions by precise control of nutrients provided to the worm. In addition, drugs and supplements may also be packed into liposomes alongside the food source for pharmacological and toxicology (LD50) studies. Our results also call for a reassessment of past findings using liquid-based diets, as we have demonstrated the particle-dependent mechanism that allows them to function.

## Supplementary Material

Supplemental material is available online at www.g3journal.org/lookup/suppl/doi:10.1534/g3.117.300325/-/DC1.
