# Supplementary material for: Growth of Caenorhabditis elegans in Defined Media Is Dependent on Presence of Particulate Matter
Source: G3 (Bethesda). 2017 Dec 8;8(2):567–75. doi: 10.1534/g3.117.300325 (PMC5919726; doi:10.1534/g3.117.300325)
Supplement: Supplementary file 1 [file 567TableS1.docx]

**TABLE S1. LIFE STAGES AT 24-HOUR INTERVALS IN TESTED DIETARY CONITIONS**

|  | **24 hours** | **48 hours** | **72 hours** | **96 hours** | **120 hours** | **144 Hours** |
| --- | --- | --- | --- | --- | --- | --- |
| **CeHR + Milk** | L1 | L2 | L4 | Adult | Adult | Adult |
| **CeHR + Particulate Mater** | L1 | L2 | L4 | Adult | Adult | Adult |
| **Milk Particulate Matter** | L1 | L2 | L3 | L4 | Adult | Adult |
| **CeHR no Milk** | L1 | L1 | L1 | L1 | L1 | L1 |
| **CeHR Filtered** | L1 | L1 | L1 | L1 | L1 | L1 |
| **M9 Buffer** | L1 | L1 | L1 | L1 | L1 | L1 |
| **S medium and OP50 (4.5 x 10^9^)** | L1 | L3 | Adult | Adult | Adult | Adult |
| **S medium and OP50 (9 x 10^8^)** | L1 | L2 | L3 | L4 | Adult | Adult |
| **Control Media** | L1 | L2 | L3 | Adult | Adult | Adult |
| **Solubilised Media** | L1 | L1 | L1 | L1 | L1 | L1 |
| **AXM** | L1 | L2 | L2 | L3 | L3 | L3 |
| **CeMM** | L1 | L2 | L2 | L3 | L3 | L3 |
| **AXM Filtered** | L1 | L1 | L1 | L1 | L1 | L1 |
| **CeMM Filtered** | L1 | L1 | L1 | L1 | L2 | L2 |
| **Liposomes packed with CeHR no Milk** | L1 | L2 | L4 | Adult | Adult | Adult |
| **Liposomes packed with M9** | L1 | L1 | L1 | L1 | L1 | L1 |
| **Liposomes packed with filtered AXM** | L1 | L2 | L4 | Adult | Adult | Adult |
| **Liposomes packed with filtered CeMM** | L1 | L2 | L4 | Adult | Adult | Adult |
| **Liposomes packed with CeHR, no milk or cholesterol** | L1 | L1 | L1 | L1 | L1 | L1 |


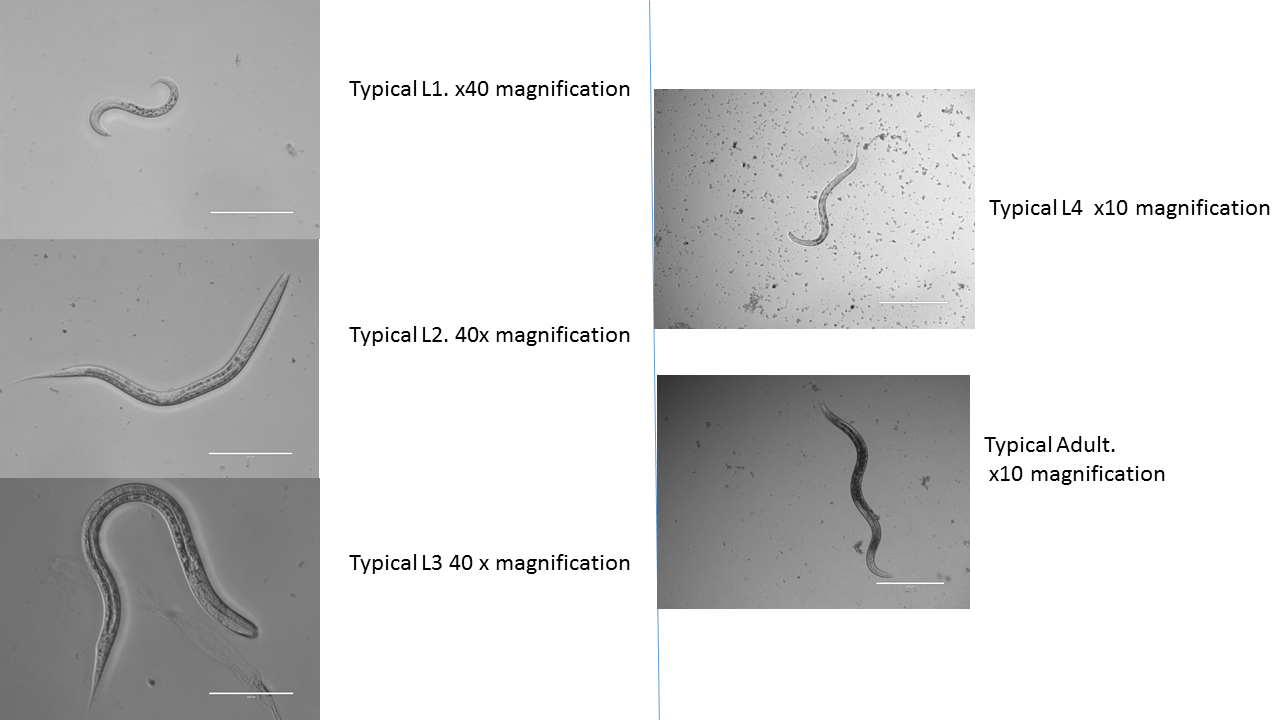


Typical worms at each life stage: Worms were scored as which of these life stages they best represented at 24 hour intervals. L1 arrest was scored when there was no change from the L1 phenotype across 144 hours.
